# Utilization of Spent FCC Catalyst as Fine Aggregate in Non-sintered Brick: Alkali Activation and Environmental Risk Assessment

**DOI:** 10.3389/fchem.2021.674271

**Published:** 2021-04-26

**Authors:** Dandan Zhang, Shiping Fang, Hongzhe Zhang, Zhengwei Liu, Zhiyuan Zhang, Shucai Zhang

**Affiliations:** ^1^State Key Laboratory of Safety and Control for Chemicals, SINOPEC Research Institute of Safety Engineering, Qingdao, China; ^2^National Registration Center for Chemicals of the State Administration of Work Safety, Qingdao, China

**Keywords:** spent FCC catalyst, reclycing, non-sintered brick, environmental risk, alkali activation

## Abstract

This study focuses on the recycling of a spent fluid catalytic cracking (FCC) catalyst to produce catalyst-based non-sintered bricks (CN-bricks) for the recovery of its aluminosilicate components and the solidification of heavy metals. The effects of the content of cement (10–20%), the proportion of FCC (10–40%), and the type of an activator (NaOH/Na_2_SiO_3_/Na_2_SO_4_) on the performance of a CN-brick were investigated in terms of the mechanical strength and leaching behavior. The results show that an optimal binder system of 20% cement + Na_2_SO_4_ could promote the compressive strength up to 42.3 MPa; the proportion of an optimal spent FCC catalyst of 20% could achieve the lowest porosity and water absorption. The microscopic mechanism of a cementitious process was analyzed by x-ray diffraction (XRD), Fourier transform infrared spectroscopy (FTIR), and scanning electron microscopy (SEM), proving that C-S-H and ettringite (AFt) are the two main hydration products of a CN-brick. Na_2_SO_4_ is superior to NaOH or Na_2_SiO_3_ as an activator since Na_2_SO_4_ takes advantage of the aluminum-rich property of a spent FCC catalyst and specifically promote the formation of a needle-like AFt. Quantitative environmental risk assessment for the utilization of a CN-brick on roads was carried out based on the leaching test of a toxicity characteristic leaching procedure (TCLP), NEN 7371 maximum availability test, and the hazard Index (HI) identification, and a final HI 0.0045 (<1.0) indicates an acceptable risk for environment and nearby residents as CN-bricks are utilized on roads for 30 years.

## Introduction

A fluid catalytic cracking (FCC) catalyst is widely used in FCC units in petrochemical industries to convert crude oil into gasoline and other lighter fuel products, and its worldwide supply can reach 840,000 tons every year (Vogt and Weckhuysen, 2015). FCC catalysts mainly consist of a clay matrix, binder, and zeolite. Due to the accumulation of heavy metals and coke on the surface, a FCC catalyst will gradually get deactivated due to the site coverage and pore blockage (Zhang et al., 2020), and the catalysts with low catalytic activity will be discharged from a work unit. A spent FCC catalyst has been identified as a hazardous waste HW50 according to the Chinese National Directory of Hazardous Wastes (HW, 2016), due to excessive contents of heavy metals [vanadium (V) and nickel (Ni)]. Nowadays, a main handling way of a spent FCC catalyst is landfilling, yet many factors will restrict this approach in the future, especially its economic costs brought by a land use right, and the environmental impact after a landfill leachate penetrates into the soil. Instead, a spent FCC catalyst has widely been explored as a replacement of the raw materials in the production (laboratory scale) of a cement mortar (Al-Jabri et al., 2013; Payá et al., 2013), a concrete (Neves et al., 2015), ceramics (Ramezani et al., 2017), a geopolymer (Font et al., 2017), a zeolite (Ferella et al., 2019), an asphalt (Xue et al., 2020), and a brick (Taha et al., 2012).

Brick is a widely used material in the construction industry, and its consumption can reach 23–30 million pieces annually in the USA alone (Meng et al., 2018). Most of the used bricks belonging to the “sintered brick” are made from clay and sintered at around 900°C, which consume a high amount of clay and energy and release a high level of CO_2_. Therefore, many countries have enacted policies to restrict the use of sintered bricks. In this case, a non-sintered brick (NS-brick) has attracted more attention because of its environmental benefit: an NS-brick is generally conserved at room temperature and hardly consumes much energy. Pozzolanic materials such as furnace slag, fly ash, iron-ore tailing, and FCC catalyst are often utilized in the production of NS-bricks, since their silica-aluminum structure can react with Ca(OH)_2_ to form a cementitious substance like calcium silicate hydrates (CSH) and calcium aluminate hydrates (CAH), contributing to the strength development. Dabaieh et al. (2020) proved that the replacement of sintering by an NS-brick can result in a reduction of the energy consumption of 5,305 MJ and the CO_2_ emission of 5,907 kg for 1,000 bricks. Wang Y. et al. (2019) adopted an electrolytic manganese residue (EMR) to produce NS-bricks. Lang et al. (2020) mixed dredged sludge, cement, lime, fly ash, and nano-SiO_2_ to produce high-strength bricks. Weishi et al. (2018) prepared an NS-brick made from iron-ore tailing and two minor additives such as triethanolamine and stearic acids. Taha et al. (2012) focuses on the recycled spent FCC catalyst to produce an NS-brick, which proved that 15% of the catalyst replacement and the addition of a cement kiln dust (CKD) can yield a higher compressive strength, showing that a spent FCC catalyst has a potential for use in road base and masonry block construction.

A spent FCC catalyst generally contains Ni and V and other heavy metals, which may pose a threat to the environment when recycled, so the kernel problem of a catalyst-based non-sintered brick (CN-brick) concerns whether their environmental risk can be acceptable when they are used on roads, embankments, and other scenarios. The study by Taha et al. (2012) have included the test of a toxicity characteristic leaching procedure (TCLP), but fails to consider in detail the extent of contaminants leached to groundwater and ingested by nearby residents through drinking water. In this study, all the processes have been considered and precisely calculated. Beyond that, the methods for improving the mechanical property of a CN-brick has not been discussed earlier. Numerous studies have included the alkali activation as an approach for the strength improvement for construction products (Guo et al., 2010; Liu et al., 2021), since an alkali environment could dissolve Si-O and Al-O from the former structure and react with Ca(OH)_2_ to produce more C-S-H and C-A-S-H gels. Alkali reagents such as NaOH, Na_2_SiO_3_, Na_2_SO_4_ all have the potential to improve the strength of a CN-brick to a higher level.

This study was designed to evaluate the utilization of a spent FCC catalyst to produce CN-bricks for use on roads and embankments. The content of cement, type of an activator, and proportion of FCC were explored for an optimal design. The compressive strength and flexural strength were tested to evaluate the physical and mechanical properties of CN-bricks. The solidification mechanism of cement and an activator was investigated by means of x-ray diffraction (XRD), scanning electron microscopy (SEM), and Fourier transform infrared spectroscopy (FTIR). In addition, quantitative environmental risk assessment for CN-bricks including a leaching test of heavy metals and an exposure scenario analysis was carried out.

## Materials and Methods

### Sample Sources

**Figure 1:**
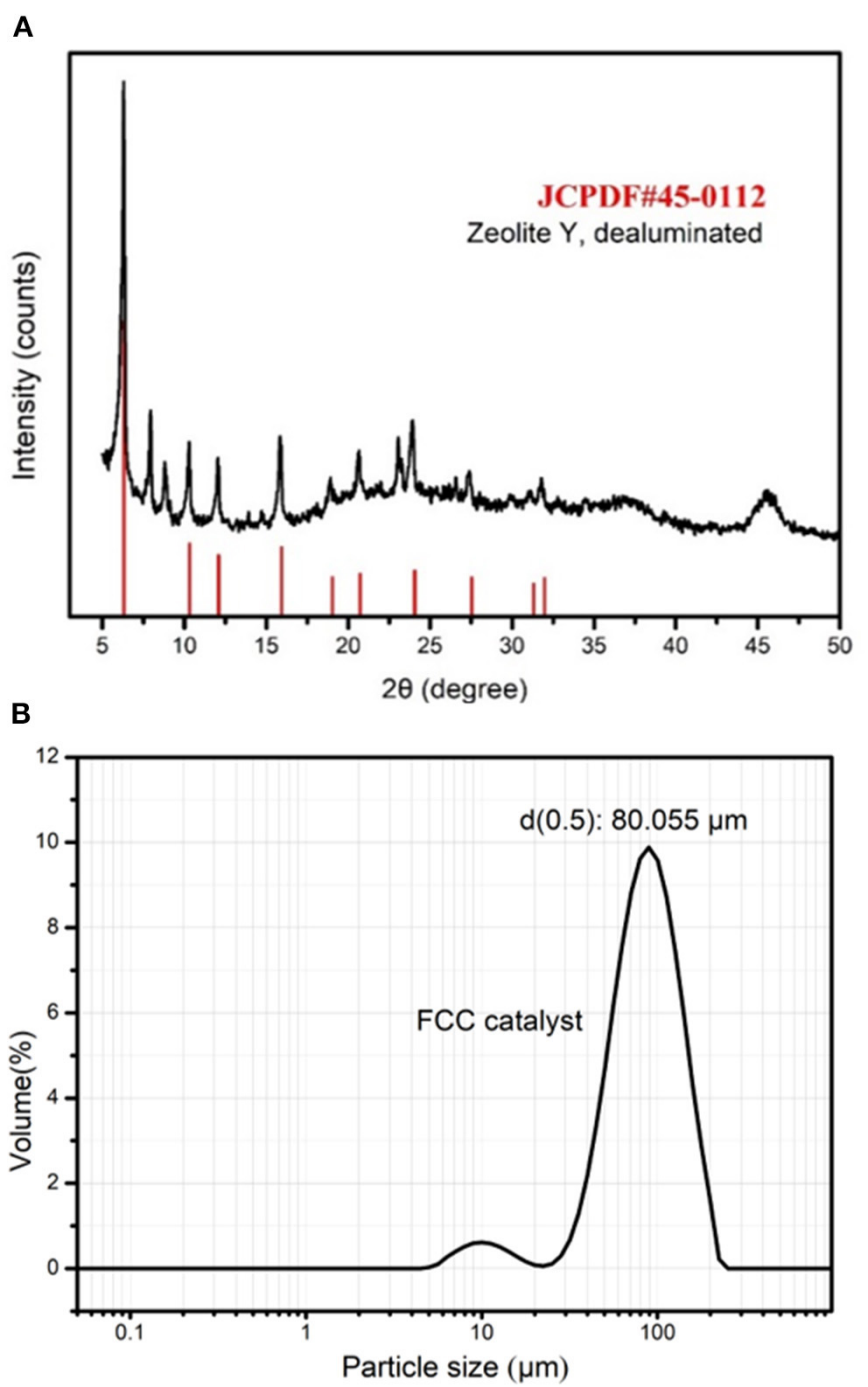
X-ray diffraction (XRD) pattern **(A)** and particle size distribution **(B)** of spent fluid catalytic cracking (FCC) catalyst.

The spent FCC catalyst sample used in this study was taken from an oil refinery in Shandong, China. X-ray fluorescence (XRF) spectrometry (Table 1) shows its major components, which are aluminum oxide (54.2%) and silicon dioxide (37.1%), and the sum of these components accounts for more than 93% of the total. The XRD pattern (Figure 1A) shows that the main phase of a spent FCC catalyst is zeolite Y, dealuminated, which is consistent with its crystal structure. **Figure 4A** shows that the surface appearance of a spent FCC catalyst is a fairly regular sphere with impurities attached to the surface. The average particle size of a spent FCC catalyst is 80.055 μm as shown in Figure 1B, which is quite low just like the other pozzolanic materials such as fly ash and furnace slag (around 20 μm).

**Table 1:** The main chemical composition of a spent fluid catalytic cracking (FCC) catalyst.

**Component**	**Al_**2**_O_**3**_**	**SiO_**2**_**	**NiO**	**V_**2**_O_**5**_**	**Sb_**2**_O_**3**_**	**Else**
Contents (wt%)	54.2	37.1	1.4	0.46	0.39	6.45

A coarse aggregate is gravel, the main (83%) particle size of a coarse aggregate was between 3 and 5 mm and the remaining (17%) was between 1 and 3 mm in diameter. The gravels were filtered through a 1.0-mm sieve, and the gravels larger than 1.0 mm were selected for use. A fine aggregate is ISO standard sand, the diameter of a fine aggregate is mainly distributed between 0.08 and 1.0 mm. The basic cementitious material is ordinary Portland P42.5 cement. In order to improve the cementitious effect of a binder, three chemical reagents such as NaOH (96% purity), Na_2_SiO_3_ · 9H_2_O, and Na_2_SO_4_ (99% purity) were sampled as the activators and were purchased from Sinopharm Company Ltd.

### Production Process of CN*-*Bricks

The production process of CN-bricks mainly includes mixing, molding, compacting, and curing. This study established 12 groups to investigate the effect of a binder system (“BS-series”) and the proportion of a spent FCC catalyst (“FC-series”) on the properties of CN-bricks, as summarized in Table 2. Samples BS-C10/C15/C20 correspond to 10, 15, and 20% content of ordinary Portland cement by weight in their raw materials. Three types of activators, NaOH, Na_2_SiO_3_, and Na_2_SO_4_, were tested for their activating function, and their samples were labeled as BS-C20A, BS-20B, and BS-20C, respectively. The additive amount of activators was set as 2.5% (wt%) referring to relevant alkali activating studies (Abdel-Gawwad et al., 2020b). Each sample of FC-10/15/20/25/30/40 represents 10, 15, 20, 25, 30, and 40% of a spent FCC catalyst by weight used in a raw material when the proportion of a gravel gets reduced from 55 to 25%.

**Table 2:** Mix composition design of a FC-series and BS-series (% by weight).

**Samples**	**Aggregate**			**Binder system**				**W/C ratio**
	**Spent FCC catalyst/%**	**Sand/%**	**Gravel/%**	**Cement/%**	**NaOH/%**	**Na_**2**_SiO_**3**_/%**	**Na_**2**_SO_**4**_/%**	
BS-C10	23	17	50	10	0	0	0	0.4
BS-C15	21	16	48	15	0	0	0	0.4
BS-C20	20	15	45	20	0	0	0	0.4
BS-C20A	20	15	45	20	2.5	0	0	0.4
BS-C20B	20	15	45	20	0	2.5	0	0.4
BS-C20C	20	15	45	20	0	0	2.5	0.4
FC-10	10	15	55	20	0	0	0	0.4
FC-15	15	15	50	20	0	0	0	0.4
FC-20	20	15	45	20	0	0	0	0.4
FC-25	25	15	40	20	0	0	0	0.4
FC-30	30	15	35	20	0	0	0	0.4
FC-40	40	15	25	20	0	0	0	0.4

In the making process of each sample, raw materials were mixed in the blender for 5 min and added with water to maintain its moisture with the water/cement ratio set as 0.40. After full blending, the materials were put in a mold and compacted with a pressure of 20 MPa to be shaped as 240 ×115 ×53 mm. After 24 h, the CN-bricks were demolded and conserved in curing boxes at 20°C and 60–90% moisture for 28 days. After 28 days, various properties of bricks were tested by the standard methods that are listed as follows.

### Mechanical and Physical Properties

The compressive strength test and flexural strength test of CN-bricks were conducted according to the Chinese Standard GB/T 4111-2013 (GB/T 4111, 2013), which were tested by Instron 5969 and Sinter WDW-50 pressure testing machines at a rate of 4.0 kN/s until the bricks got damaged. The bulk density and water absorption were also tested according to GB/T 4111-2013 by soaking the samples in a tank at 20°C for 24 h when the upper surface was at least 20 mm lower than the water surface, and then the samples were taken out, wiped, and immediately weighed as *m*_1_. Afterward, the samples were dried in an oven at 105°C to achieve constant weight and weighed as *m*. The water absorption was calculated as follows:

(1) $$\begin{array}{r} \text{Water absorption}=\frac{{\text{m}}_{1}-\text{m}}{\text{m}}\times 100\% \end{array}$$

The bulk density was calculated according to Equation (2)

(2) $$\begin{array}{r} \text{Bulk density }\left(\text{kg}/{\text{m}}^{3}\right)=\frac{\text{m}}{\text{V}} \end{array}$$

where *m* stands for the dry weight of CN-bricks and *V* is the volume of a brick. Each index was measured thrice and reported with error bars in the final result.

### Microstructure Characterization

For subsequent microstructure analysis, the chosen samples were roughly crushed and pulverized in a laboratory grinding machine for 5 min, and then dried in an oven at 50°C until reaching a constant weight. The samples that could pass through a 0.45-μm filter were chosen for the XRD test and FTIR test. XRD analysis was carried out to obtain the crystal structure and phase characteristics of a CN-brick on a Bruker D8 Advance with the scanning angle ranging from 5 to 75° at a scanning rate of 4°/min. FTIR analysis was carried out to identify the functional groups of organic compounds on a Thermo Nicolet iS5 in the range of 4,000–500 cm^−1^. The crushed samples were analyzed by SEM to study the microstructure of the specimens on a Tescan Mira 3 machine.

### Environmental Risk Assessment of CN*-*Bricks

#### Determination of Total Metal Content

The detection of the total heavy metal content of heavy metals V, chromium (Cr), manganese (Mn), cobalt (Co), Ni, Cu, Zn, antimony (Sb), Ba, and Pb in raw materials and CN-bricks was done by following the Chinese Standard HJ 781-2016 (HJ 781, 2016). To sum up, the solid wastes were added with acids including HCL, HF, HNO_3_, and HClO${}_{4}^{,}$ and then digested in an electric hot plate at 180°C for 1–2 h. Digested solution was then brought into plasma emission spectrometer [inductively coupled plasma optical emission spectroscopy (ICP-OES), Thermo iCAP 7000], and the characteristic spectrum of elements being atomized will be used to represent the element content.

#### Toxicity Characteristic Leaching Procedure

The TCLP test was conducted under a protocol EPA SW-846 Method 1311 (EPA 1311, 1992) to simulate a leaching process induced by acid rain when CN-bricks are used in a pedestrian street. In short, samples were mixed with the leaching solution, which contains acetic acid solution (0.57% v/v) with a liquid-to-solid (L/S) ratio set as 20:1 L/kg and pH set as 2.88 ± 0.05. The mixed solution was then vibrated for 18 h under a temperature of 23°C at a rate of 30 revolutions/min. The leachate was then filtered and analyzed by ICP-OES to determine the leaching concentration of heavy metals V, Cr, Mn, Co, Ni, Cu, Zn, Sb, Ba, and Pb.

#### Maximum Availability Leaching Test

Heavy metals V, Cr, Co, Ni, Sb, and Ba are tested for their maximum availability in CN-bricks according to the EA NEN 7371 protocol (EA NEN 7371, 2004), which includes two extraction stages. Samples were filtered through a 125 μm membrane and mixed with the extract liquid at an L/S ratio of 50:1 L/kg, with pH maintained at 7.0 ± 0.5 by adjusting HNO_3_ in deionized water. After the first extraction, the leachate was filtered through 0.45 μm and be prepared for second extraction stage, during which the pH is changed to 4.0 ± 0.5. The contact time was set as 3 h for both extraction process. At last, the two leachates were mixed and analyzed by ICP-OES. The maximum leaching rate is calculated in Equation (3):

(3) $$\begin{array}{r} \text{Maximum leaching rate }\left(\text{\%}\right)=\frac{\text{Maximum availability}}{\text{Total metal content}} \end{array}$$

## Results and Discussion

### Mechanical and Physical Properties of CN-Bricks

#### Compressive Strength and Flexural Strength

**Figure 2:**
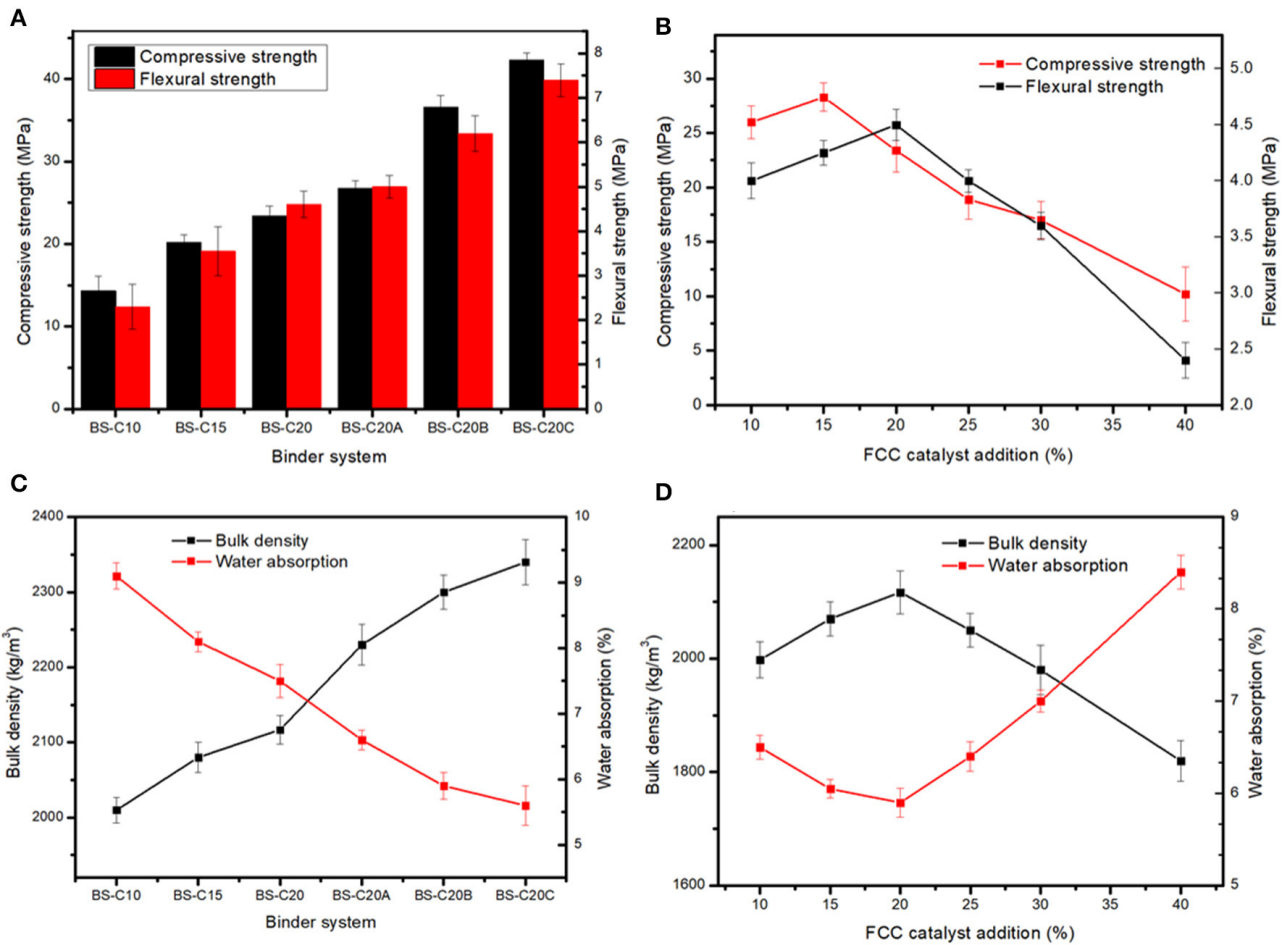
Compressive strength, flexural strength **(A,B)** water absorption, and bulk density **(C,D)** of catalyst-based non-sintered bricks (CN-bricks).

The effect of the cement content and alkali activators on the mechanical properties of a CN-brick was examined. A review of Gupta et al. (2020) illustrates that cement (10–30%) and lime (5–30%) are the main cementitious binders used in unfired bricks/blocks. In this study, 10, 15, and 20% of the cement were tested, and the compressive strength increases from 14.3 to 23.4 MPa, flexural strength increases from 2.3 to 4.6 MPa as the cement content increases from 10 to 20% (Figure 2A). The main components of Portland cement are 3CaO · SiO_2_ (C_3_S), 2CaO · SiO_2_ (C_2_S), and 3CaO · Al_2_O_3_ (C_3_A), which could react with H_2_O to form C-S-H, C-A-H, ettringite (AFt), and Ca(OH)_2_ to improve the strength of a brick, and the mechanisms of the reaction are shown in Equations (4)–(7):

(4) $$\begin{array}{r} 3\text{CaO}\cdot \text{Si}{\text{O}}_{2}+\text{n}{\text{H}}_{2}\text{O}\to \text{xCaO}\cdot \text{Si}{\text{O}}_{2}\cdot \text{y}{\text{H}}_{2}\text{O}+\left(3-\text{x}\right)\text{Ca}{\left(\text{OH}\right)}_{2} \end{array}$$

(5) $$\begin{array}{r} 2\text{CaO}\cdot \text{Si}{\text{O}}_{2}+\text{n}{\text{H}}_{2}\text{O}\to \text{xCaO}\cdot \text{Si}{\text{O}}_{2}\cdot \text{y}{\text{H}}_{2}\text{O}+\left(2-\text{x}\right)\text{Ca}{\left(\text{OH}\right)}_{2} \end{array}$$

(6) $$\begin{array}{r} 3\text{CaO}\cdot \text{A}{\text{l}}_{2}{\text{O}}_{3}+6{\text{H}}_{2}\text{O}\to 3\text{CaO}\cdot \text{A}{\text{l}}_{2}{\text{O}}_{3}\cdot 6{\text{H}}_{2}\text{O} \end{array}$$

(7) $$\begin{array}{r} 3\text{CaO}\cdot \text{A}{\text{l}}_{2}{\text{O}}_{3}+3\text{CaS}{\text{O}}_{4}\cdot 2{\text{H}}_{2}\text{O}\\ +26{\text{H}}_{2}\text{O}\to 3\text{CaO}\cdot \text{A}{\text{l}}_{2}{\text{O}}_{3}\cdot 3\text{CaS}{\text{O}}_{4}\cdot 32{\text{H}}_{2}\text{O}\left(\text{AFt}\right) \end{array}$$

A higher content of the cement indicates a higher content of hydration products that would wrap an aggregate more tightly, which then indicates a lower porosity, denser structure, and higher mechanical strength. According to the relevant national standard GB 11945-1999 (GB 11945, 1999), the compressive strength and flexural strength of the MU20-degree brick should exceed 20.0 and 4.0 MPa. Hence, only the cement content of 20% could achieve the MU20 degree. To further improve the mechanical property of a brick, alkali activators were introduced into a binder system. Figure 2 shows that NaOH slightly increases the compressive strength of a brick from 23.4 to 26.7 MPa, while Na_2_SiO_3_ and Na_2_SO_4_ significantly lift the compressive strength up to 36.6 and 42.3 MPa, respectively. Na_2_SO_4_ is an optimal activator for a CN-brick, the mechanism of which will be illustrated later in detail.

With the determination of an optimal binder system, the effect of the FCC content on the mechanical properties were then evaluated. Among raw materials, gravels (1.0–5.0 mm) act as a skeleton, sands (0.08–1.0 mm) can fill the pores between the gravels, and a spent FCC catalyst (80.1 μm) can fill the remaining interspace. An ideal grain size distribution can reduce the overall porosity and induces a higher strength. Figure 2 shows 20% FCC proportion could achieve the most ideal compressive strength and flexural strength at the same time, the strength at this point can meet the MU20 class according to the Chinese standard GB 11945-1999, yet up to 25% addiction amount will reduce the strength of bricks to MU15 class. Flexural strength changes in the same trend as compressive strength, the cement content <20% could improve the flexural strength, but a proportion higher than 20% would deteriorate the strength of a CN-brick, the mechanism of which will be illustrated later. In sum, an optimal formula of the production of a CN-brick is gravel:sand:FCC:cement:Na_2_SO_4_ = 45:15:20:20:2.5.

#### Water Absorption and Bulk Density

The water absorption and bulk density of CN-bricks with various binders and the FCC content are summarized in Figure 2. The bulk density of a CN-brick increases from 2,010 to 2,340 kg/m^3^ when the cement content increases from 10 to 20%, and then added with three types of activators. This is because cement and activators increased the amount of hydration products like C-S-H and AFt, as illustrated by XRD and FTIR in later sections, which could fill the micropores of an aggregate and bind them more tightly. This is also the reason why the water absorption of BS-series decreases gradually from 9.1 to 5.6%. The water absorption represents the water-resistance ability of bricks that is closely related to the porosity of bricks. When aggregates are bound more tightly by cement and activators, open porosity will decrease, so does the water absorption value (Lang et al., 2020). Even though the water absorption rates of samples are different, all are below the threshold set by the Chinese standard JC/T 422-2007, which is 18%.

The bulk density and water absorption of a CN-brick with different values of FCC content are illustrated in Figure 2D. The bulk density increases when the proportion of FCC gets increased from 10 to 20% and the content of gravel decreases from 55 to 45%, this is because macropores between the gravels (1–5 mm) could be filled more tightly by a fine aggregate of a spent FCC catalyst (0.08 mm), which reduces the porosity and improves the compactness, so the water absorption rate of a CN-brick decreases from 6.5 to 5.9%. However, when more FCCs are added from 25 to 40% and the content of gravel gets reduced from 40 to 25%, a spent FCC catalyst replaces gravels as a main raw material in a system, the properties of a CN-brick get deteriorated. The bulk density of bricks gradually decreases from 2,117 to 1,820 kg/m^3^ accompanied with the compressive strength from 23.4 to 10.2 MPa (Figure 2B), while the water absorption rate increases from 5.9 to 8.4%. A higher proportion of fine aggregates than coarse aggregates would induce an incompact grain size distribution. Fine aggregates that could not be compacted by large particles would be scattered in the system. The above mentioned research shows that too much coarse aggregates or fine aggregates are neither good for the properties of bricks, 20% of a spent FCC catalyst and 45% of a gravel could achieve the most ideal stacking structure in a CN-brick.

### Microstructure of CN-Bricks Under Alkali Activation

#### XRD Characterization

**Figure 3:**
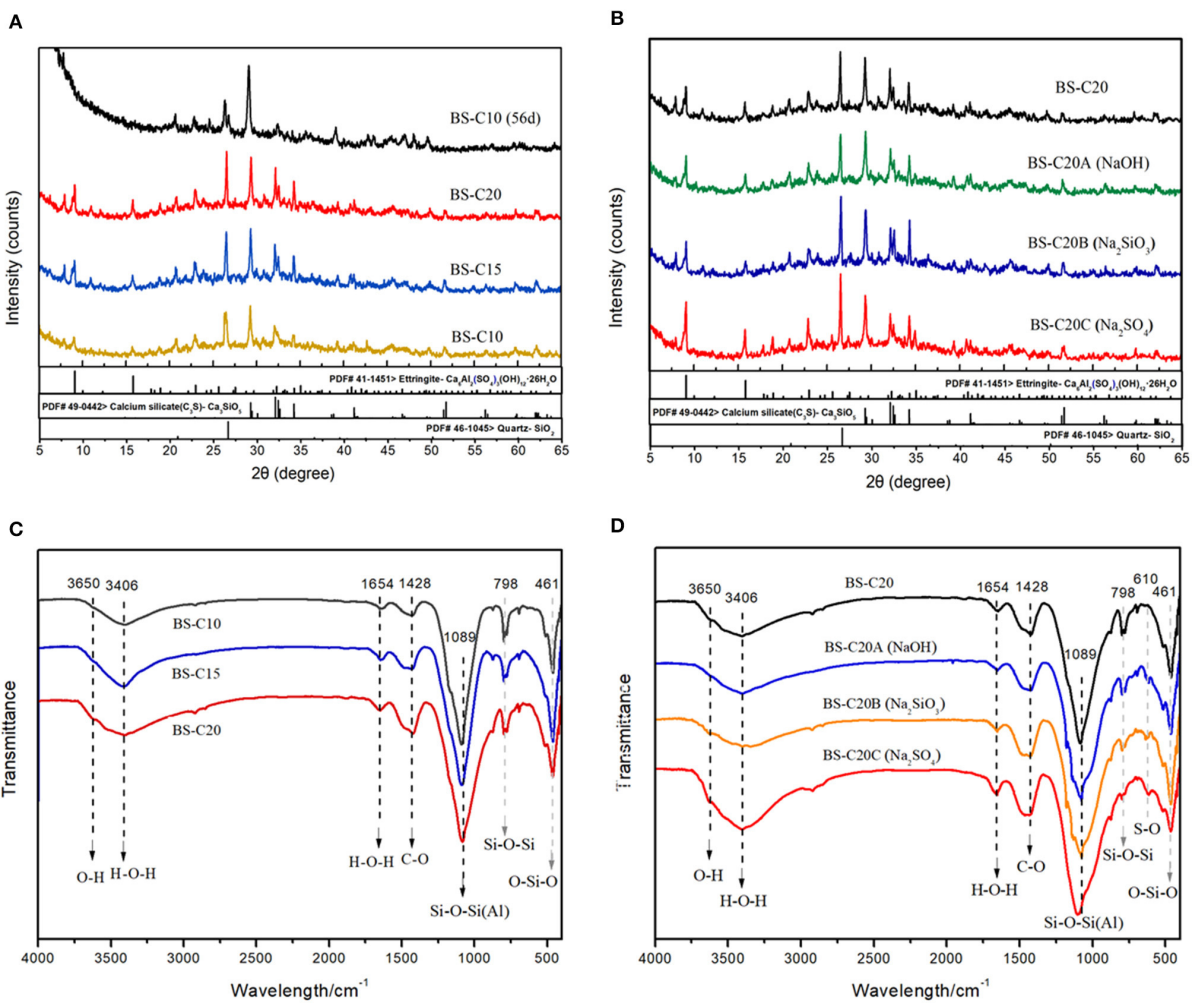
XRD **(A,B)** and Fourier transform infrared spectroscopy (FTIR) patterns **(C,D)** of CN-bricks (BS-series).

The mechanical strength of a CN-brick is mainly attributed by the hydration reaction caused by binders including cement, NaOH, Na_2_SiO_3_, and Na_2_SO_4_, in this study. Figure 3A illustrate the XRD pattern of CN-bricks added with 10, 15, and 20% cement (BS-C10, BS-C15, and BS-C20) at 28 days and 10% cement at 56 days. It can be seen that the crystalline phase in the CN-bricks are AFt (9.1, 15.8, 22.9, 34.5, 40.8°, 2θ), quartz (SiO_2_, 26.5°), C_3_S (Ca_3_SiO_5_, 29.2°, 32.1°, 34.2°, 2θ), C_2_S (Ca_2_SiO_4_, 32.0°, 32.5°, 34.3°, 2θ), and C-S-H (29.3°, 32.0°, 50.0°, 2θ). The two hydration products with higher peaks that assume a main responsibility for the strength of CN-bricks are AFt and C-S-H. These two products can be formed by ordinary Portland cement alone as illustrated in Equations (5–8). Apart from that, they can also be formed by a chemical reaction between an aggregate and cement. The Ca(OH)_2_ produced from the cement hydration (Equations 4 and 5) could react with SiO_2_ and Al_2_O_3_ that exists in a gravel, sand, and a spent FCC catalyst, and then form C-S-H and AFt directly according to Equations (8) and (9):

(8) $$\begin{array}{r} 3\text{Ca}(\text{OH}{)}_{2}+2\text{Si}{\text{O}}_{2}\to 3\text{CaO}\cdot 2\text{Si}{\text{O}}_{2}\cdot 3{\text{H}}_{2}\text{O} \end{array}$$

(9) $$\begin{array}{r} 3\text{Ca}(\text{OH}{)}_{2}+\text{A}{\text{l}}_{2}{\text{O}}_{3}+3\text{CaS}{\text{O}}_{4}\cdot 2{\text{H}}_{2}\text{O}\\ +26{\text{H}}_{2}\text{O}\to 3\text{CaO}\cdot \text{A}{\text{l}}_{2}{\text{O}}_{3}\cdot 3\text{CaS}{\text{O}}_{4}\cdot 32{\text{H}}_{2}\text{O} \end{array}$$

An interesting phenomenon in the XRD pattern of a CN-brick is that the crystalline phase of AFt is far more distinct compared with the bricks made from the other wastes, such as fly ash, furnace slag, or degraded sludge (Wang C.-Q., et al., 2019; Akinyemi et al., 2021). This is because of the proportion of Al_2_O_3_ in a spent FCC catalyst, 54.2%, which is significantly higher than that of fly ash (18–25%), furnace slag (6–11%), and degraded sludge (7–21%) and it is referenced in many relevant literature studies. Multiple researches related to the spent FCC catalyst recycling (Al-Jabri et al., 2013; Font et al., 2017; Xue et al., 2020) also recorded that the proportion of Al_2_O_3_ in a spent FCC catalyst ranges between 47 and 66%, which is in a relatively high level. A higher content of Al_2_O_3_ would react with Ca(OH)_2_, and then form more AFt than the other materials, during which the content of CaSO_4_ is a limiting factor.

With an increase in the proportion of cement from 10 to 20%, all of the peaks have increased including SiO_2_, C_3_S, C_2_S, C-S-H, and AFt (Figure 3A). This is because of the cement hydration itself illustrated in Equations (5–8). Figure 3A also compares the XRD pattern of BS-C10 at 28 and 56 days. Some crystalline phases like SiO_2_, C_3_S, and C_2_S get decreased and the peaks of C-S-H (29.3°, 50.0°, 2θ) have increased in BS-C10 (56 days). Hydration products like C-S-H and AFt could be continuously produced as the curing time gets prolonged, yet 28 days are enough for the mechanical construction in a CN-brick.

The XRD pattern of BS-C20A/B/C is shown in Figure 3B. Compared to BS-C20, crystalline phases of AFt and C-S-H in BS-20A show a slight improvement. NaOH could dissolve [SiO_4_]^4−^ and [AlO_4_]^5−^ from a spent FCC catalyst and aggregates, and promote their reaction with Ca^2+^ to produce more C-S-H and C-A-H gels. Compared to BS-C20A, the peaks of SiO_2_ and C_3_S in a sample BS-C20B were obviously increased, since Na_2_SiO_3_ could produce SiO_2_, CaSiO_3_, and NaOH as shown in Equation (10):

(10) $$\begin{array}{r} \text{N}\text{aO}\cdot \text{nSi}{\text{O}}_{\text{2}}\text{+Ca(OH}{\text{)}}_{\text{2}}\to \text{(n-1)Si}{\text{O}}_{\text{2}}\text{+CaSi}{\text{O}}_{\text{3}}\text{+2NaOH} \end{array}$$

The increased content of SiO_2_ reacts with Ca(OH)_2_ to produce more C_3_S and C-S-H as XRD results show, thus increasing the compressive strength of a CN-brick. In addition, the generated NaOH will dissolve Si-O and Al-O and promote the formation of C-S-H and C-A-H at the same time like the BS-C20A does.

When Na_2_SO_4_ are added into BS-C20C, significant increases in the peak heights of AFt are at 9.1, 15.8, and 22.9°. This is because of bringing more SO${}_{4}^{2-}$ in the system, and more CaSO_4_ and NaOH can be formed in Equation (11):

(11) $$\begin{array}{r} \text{N}{\text{a}}_{2}\text{S}{\text{O}}_{4}+\text{Ca}(\text{OH}{)}_{2}\to \text{CaS}{\text{O}}_{4}\cdot 2{\text{H}}_{2}\text{O}+2\text{NaOH} \end{array}$$

It is mentioned above that AFt is a characteristic hydration product of a CN-brick, which is transformed from a high content of Al_2_O_3_, during which CaSO_4_ is a limiting factor. The addiction of an activator Na_2_SO_4_ could promote the formation of CaSO_4_ · 2H_2_O and then react with excessive Al_2_O_3_ to increase the content of AFt as the XRD results show. This is a main reason why Na_2_SO_4_ could increase the compressive strength of a CN-brick from 23.4 to 42.3 MPa. This rule has not been found in the previous studies on the recycling of a spent FCC catalyst. Na_2_SO_4_ is more suitable to be an activator in a NS-bricks made from a spent FCC catalyst.

#### FTIR Analysis

The FTIR pattern of BS-C10/15/20 and BS-C20A/B/C is summarized in Figures 3C,D. These absorption peaks mainly represent the stretching O-H vibration (3,650 cm^−1^), stretching H-O-H vibration in water (3,406 and 1,654 cm^−1^), asymmetric stretching CO${}_{3}^{2-}$ vibration (1,428 cm^−1^), asymmetric stretching Si-O-Si(Al) bond (1,098 cm^−1^), and bending O-Si-O vibration (461 cm^−1^) in a gel matrix. The predominant peaks shown in the spectra mainly refer to silicate, aluminate, and carbonate compounds just like the other construction materials made from industrial wastes and binders (Abdel-Gawwad et al., 2020a; Liu et al., 2021). When the cement content increases from 10 to 20%, there is an increase in the intensity of peak in 3,650 cm^−1^, which indicates that more Si-OH and Al-OH are produced from [SiO_4_]^4−^ and [AlO_4_]^4−^. Absorption of peaks in 3,406 and 1,654 cm^−1^ also shows an increase in intensity, which indicates that more free water has been transferred to structural water. The absorption peak of Si-O-Si(Al) is located between 1,150 and 1,008 cm^−1^, which has been apparently sharpened since more Si-O are produced in the form of C-S-H and AFt, which is consistent with the result of XRD. The absorption peak at 1,428 cm^−1^ is associated with CO${}_{3}^{2-}$ existing in carbonates like calcite and dolomite, which has been increased because of the reaction of Ca^2+^ and atmospheric CO_2_. The intensity of 798 cm^−1^ peak has been decreased since more SiO_2_ has been involved in the hydration process. A higher cement content is proved to be effective in promoting the hydration process.

The FTIR pattern of CN-bricks added with NaOH, Na_2_SiO_3_, and Na_2_SO_4_ as activators are summarized in Figure 3D. Many peak intensities have increased when various activators are added including bands at 3,650, 1,654, 1,428, 1,089, and 461 cm^−1^. Peaks near 3,650 cm^−1^ indicates the extent of Si-OH and Al-OH dissolution rate from [SiO_4_]^4−^ and [AlO_4_]^4−^, Na_2_SiO_3_, and Na_2_SO_4_ exhibits higher dissolving ability than NaOH, which explains why Na_2_SiO_3_ and Na_2_SO_4_ exhibit higher strength enhancement capacity. The increase of band 1,428 cm^−1^ intensity (CO${}_{3}^{2-}$) indicates that activators promote the formation of carbonates and improve the strength of a brick. The absorption peak at 798 cm^−1^ (SiO_2_) gradually disappears when activators are added, which represents the extent to which SiO_2_ gets transformed to C-S-H and C-A-S-H gel. The enhancement of Si-O-Si(Al) bands at 1,098 cm^−1^ and a slight shift toward higher wavenumbers indicate the polymerization reaction and the increased amount of C-S-H and C-A-H in hydration products. The absorption peak near 610 cm^−1^ represents the bond of S-O in SO${}_{4}^{2-}$, which has been sharpened in BS-C20C compared to BS-C20, due to an addictive amount of SO${}_{4}^{2-}$ and AFt brought from an activator Na_2_SO_4_. In sum, FTIR spectrums prove that a higher content of cement and alkali activators could promote the formation of C-S-H, AFt, and carbonates in hydration products.

#### SEM Analysis

**Figure 4:**
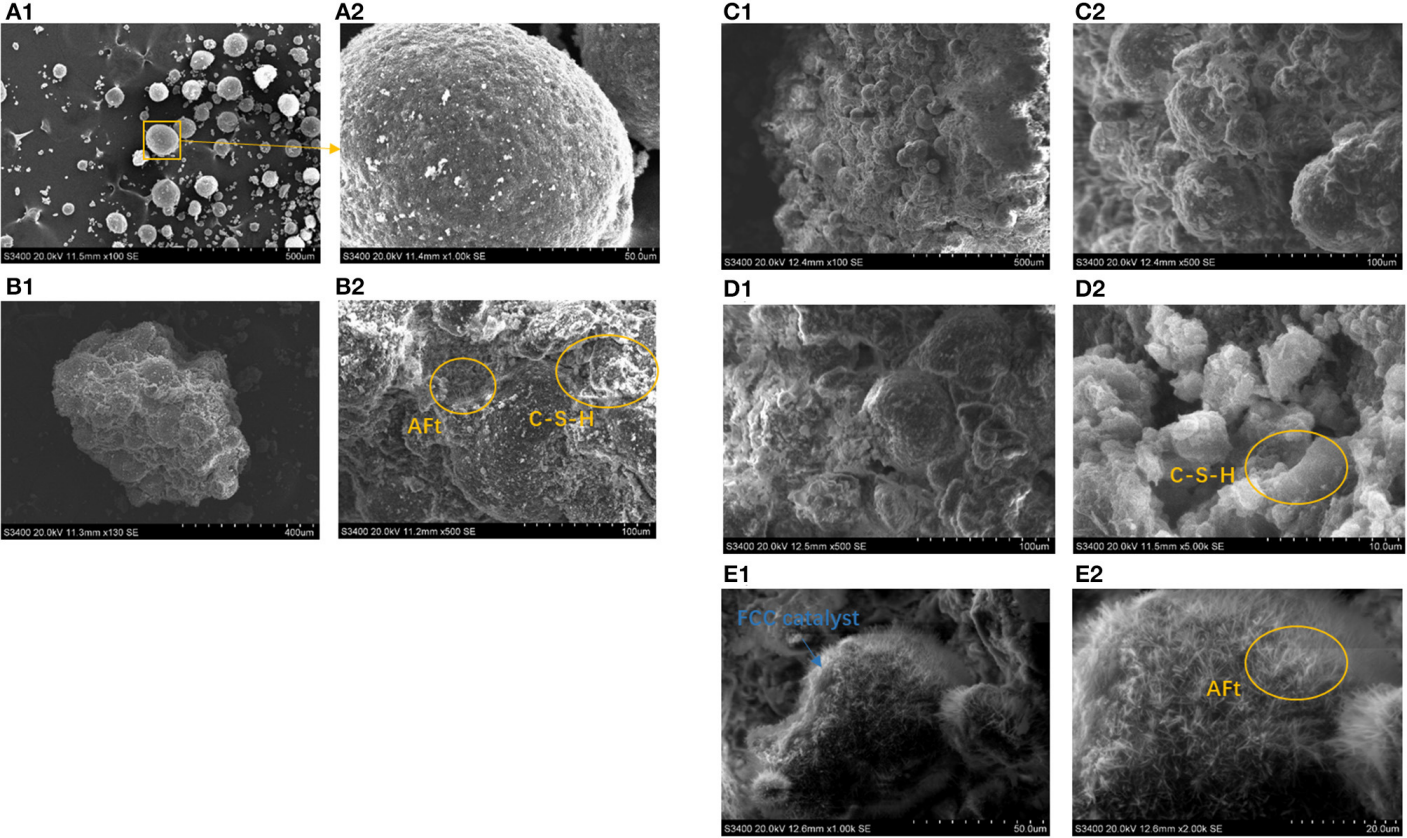
Scanning electron microscopy (SEM) images of **(A)**: spent FCC catalyst samples; **(B)** BS-C20; **(C)**: BS-C20A (NaOH); **(D)** BS-C20B (Na_2_SiO_3_); **(E)** BS-C20C (Na_2_SO_4_).

The scanning electron microscopy test was conducted for a more intuitive observation of the effect of a binder system on a CN-brick. Figure 4 shows that spent FCC catalysts are regular spheres with particle size lower than 100 μm, attached with certain impurities (around 1 μm) that are likely to be the chemical compounds produced from heavy metals in crude oil. These heavy metals would cover the exterior surface of a spent FCC catalyst and clog the access to active sites inside the zeolite, causing a spent FCC catalyst to be deactivated. When a spent FCC catalyst is recycled and reused in a CN-brick, it is tightly wrapped in the gel materials along with the aggregates as a whole (Figure 4B). The gel materials that bind them together are mainly flake-like C-S-H and needle-like AFt, which are consistent with the XRD results. AFt is usually found in a system added with gypsum, and is recorded to have a setting retarding effect for the too quick hydration speed of cement caused by C_3_A. AFt is also widely known as capable of improving early stage strength of the construction materials.

The effect of activators on hydration products is examined by SEM as shown in Figure 4. When NaOH is added into the system, the microstructure of BS-20A is generally similar to that of BS-20, with a slight difference that the surface of aggregates and a spent FCC catalyst are little bit rougher than the original one. About 3% addition of NaOH creates an alkali environment that would corrode the aluminosilicate material, dissolve more activated Si-O unit from a Si-O-Si network, and form more C-S-H gels. When Na_2_SiO_3_ is added into a system (Figure 4D), the interface of aggregate and gel materials is more compact and tight. A main gel material in the BS-C20B system is still a flake-like C-S-H, the density of which is obviously increased, since the addition of Na_2_SiO_3_ introduces a higher content of SiO_2_ and CaSiO_3_ and further a higher content of a hydration product C-S-H. When Na_2_SO_4_ is added into a binder, as shown in Figure 4E, the surface of a spent FCC catalyst and its interface with other aggregates are covered with a thick layer of a needle-like AFt, since SO${}_{4}^{2-}$ in an activator would react with Al_2_O_3_ to form more AFt (XRD result). The amount of AFt formed on the FCC surface is higher than on the surface of gravel and sand, proving that Na_2_SO_4_ could specifically activate an aluminum-rich material. Among NaOH, Na_2_SiO_3_, and Na_2_SO_4_, the activator Na_2_SO_4_ can specifically make full use of a characteristic of the FCC catalyst as a high-aluminum material.

**Figure 5:**
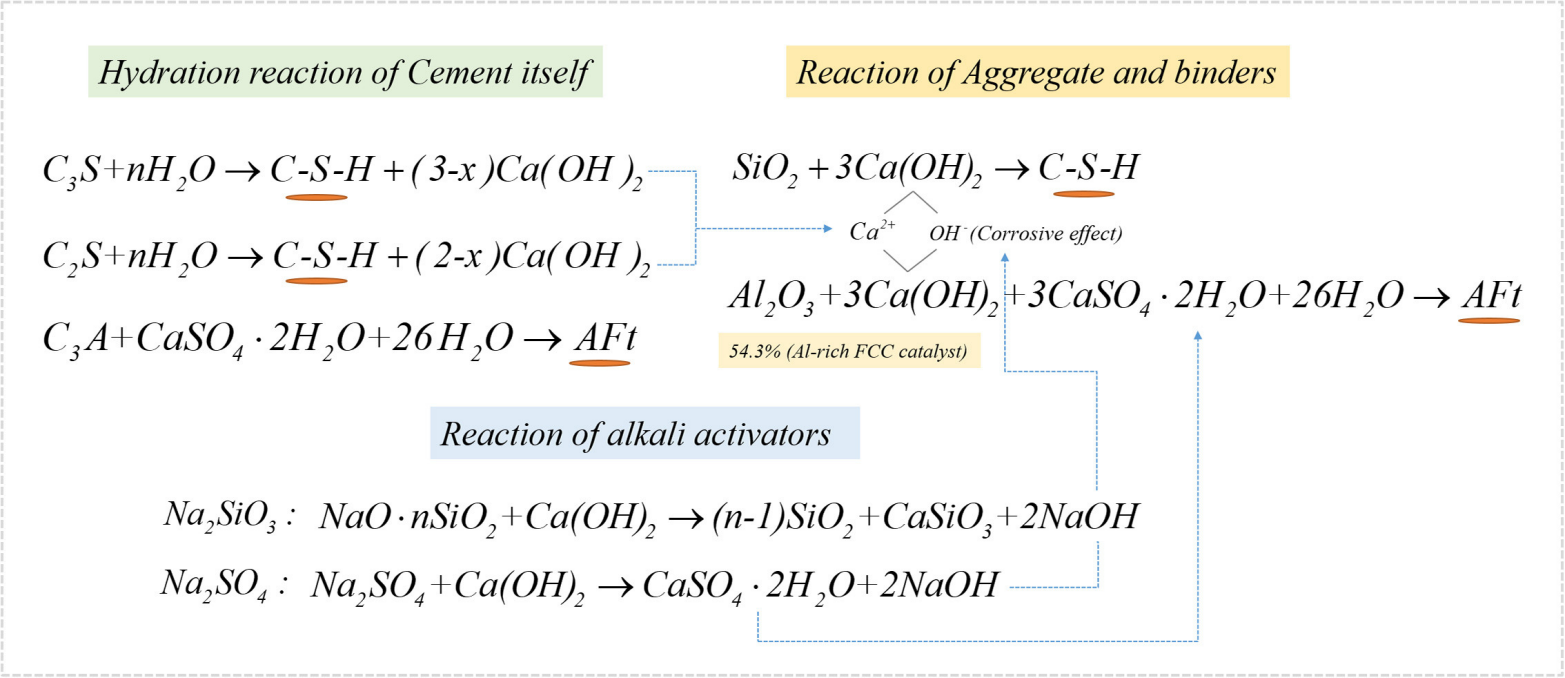
Microscopic mechanism diagram of a CN-brick.

In summary, the mechanism of hydration reactions happened in a CN-brick is illustrated in Figure 5. C-S-H and AFt are the main hydration products generated from cement, reinforced by activators, and mainly contribute to the mechanical strength of CN-bricks.

### Quantitative Environmental Risk Assessment

#### Determination of Characteristic Pollutants of CN-Bricks

**Figure 6:**
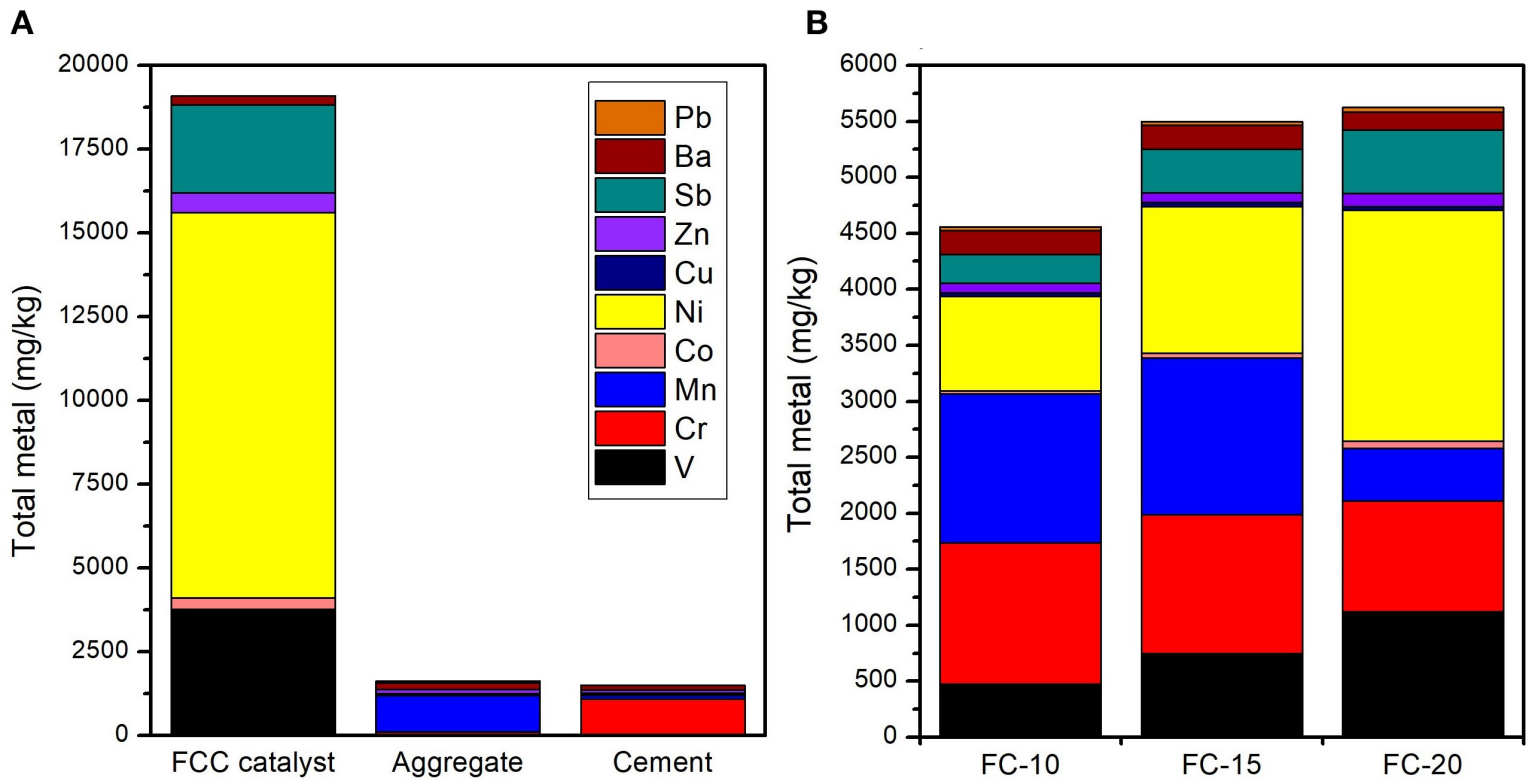
Total metal content of raw materials **(A)** and CN-bricks **(B)**.

The characteristic pollutants in CN-bricks were determined from the total metal content (Figure 6) and leaching concentration (**Table 4**). Metals V, Cr, Mn, Co, Ni, Cu, Zn, Sb, Ba, and Pb were chosen to be examined in raw materials and CN-bricks, since they are conventional indicators in Chinese hazardous waste identification standard (GB 5085.3, 2007) and groundwater quality standard (GB/T 14848, 2017). Figure 6A shows that the concentration of Ni, V, and Sb is particularly higher than the other heavy metals in a spent FCC catalyst, which are 11,510.5, 3,700.0, and 2,610.2 mg/kg, respectively. Ni and V have been widely acknowledged as the main pollutants of a FCC catalyst (Etim et al., 2016; Ferella et al., 2016). The deposition of Ni and V from crude oil on the catalyst surface makes the selectivity of the catalyst worse and leads to catalyst poisoning. V is the most dangerous metal since V in V^5+^ state reacts to form H_3_VO_4_, which could destroy the structure of zeolite dramatically (Busca et al., 2014). Ni is less detrimental, but still has a strong dehydrogenation activity, which would suppress the production of gasoline. One of the solutions is the addition of Sb to from Ni-Sb alloys, Ni_x_Sb_y_O_z_, to inhibit the strong dehydrogenation activity of Ni and increase the yield of gasoline (Bohmer et al., 1990), so Sb is in high content. Wai et al. (1999) also confirmed that the concentration of V, Ni, and Sb gets significantly increased from 2–33 ppm to 1,326–3,930 ppm when a FCC catalyst gets deactivated in an unit. Apart from the spent FCC samples, the raw material aggregates (gravel and sand) and cement also brought in a high level of Mn and Cr in the product. Elements Ni, V, Sb, Mn, and Cr are the pollutants with a high total content in CN-bricks.

Instead of the total content, the leaching behavior is usually a main concern of the hazard identification. Samples “FC-10,” “FC-15,” and “FC-20” were analyzed to investigate the leaching behavior of CN-bricks with different amounts of a spent FCC catalyst. TCLP test (Table 3) show that the leaching concentration of all metals is significantly lower than their total content, attributed by the above-mentioned hydration reaction. The regulatory level of relevant metals in China and USA are also listed in Table 3 according to the Chinese Standard (GB 5085.3, 2007) and USA 40 CFR 261.24 (USEPA, 2011), which confines the leaching concentration limits above which the solid waste will be defined as hazardous wastes. The leaching concentration of all heavy metals are much lower than the regulatory levels, thus the CN-bricks produced in this study should not be considered as hazardous materials.

**Table 3:** Toxicity characteristic leaching procedure (TCLP) result of catalyst-based non-sintered bricks (CN-bricks; ug/L).

**Samples**	**V**	**Cr**	**Mn**	**Co**	**Ni**	**Cu**	**Zn**	**Sb**	**Ba**	**Pb**
FC-10	3.34 ± 0.82	6.12 ± 0.00	ND	0.13 ± 0.01	1.20 ± 0.11	0.35 ± 0.01	6.22 ± 1.51	13.20 ± 0.63	126.07 ± 15.79	0.4 ± 0.06
FC-15	6.94 ± 0.62	6.05 ± 0.23	ND	0.11 ± 0.01	0.76 ± 0.12	0.38 ± 0.18	1.23 ± 0.96	21.26 ± 0.38	52.03 ± 3.13	0.05 ± 0.01
FC-20	78.68 ± 4.78	40.26 ± 5.70	ND	0.55 ± 0.02	0.71 ± 0.10	0.60 ± 0.10	1.12 ± 0.87	55.46 ± 1.27	19.32 ± 0.89	ND
Regul limit 1	N.	5,000	N.	N.	5,000	100,000	100,000	N.	100,000	5,000
Regu limit 2	N.	5,000	N.	N.	N.	N.	N.	N.	100,000	5,000
LOD	0.01	0.01	0.02	0.005	0.01	0.01	0.006	0.01	0.004	0.05

*ND, Not detected*.

*N., Not mentioned in reference level regulations*.

*“Regulatory limit 1” is referred to Chinese Standard GB 5085.3 (GB 5085.3, 2007)*.

*“Regulatory limit 2” is referred to the USA standard 40 CFR 261*.

*LOD, limit of detection*.

*Each data are based on three replicates*.

In the long-term environmental risk of CN-bricks, six metal elements V, Cr, Co, Ni, Sb, and Ba are identified as the characteristic pollutants of CN-bricks according to the result of TCLP, since the other elements Mn, Cu, Zn, and Pb are either in low concentration or pose a little threat to human health.

#### Impact of CN-Bricks on Groundwater

**Figure 7:**
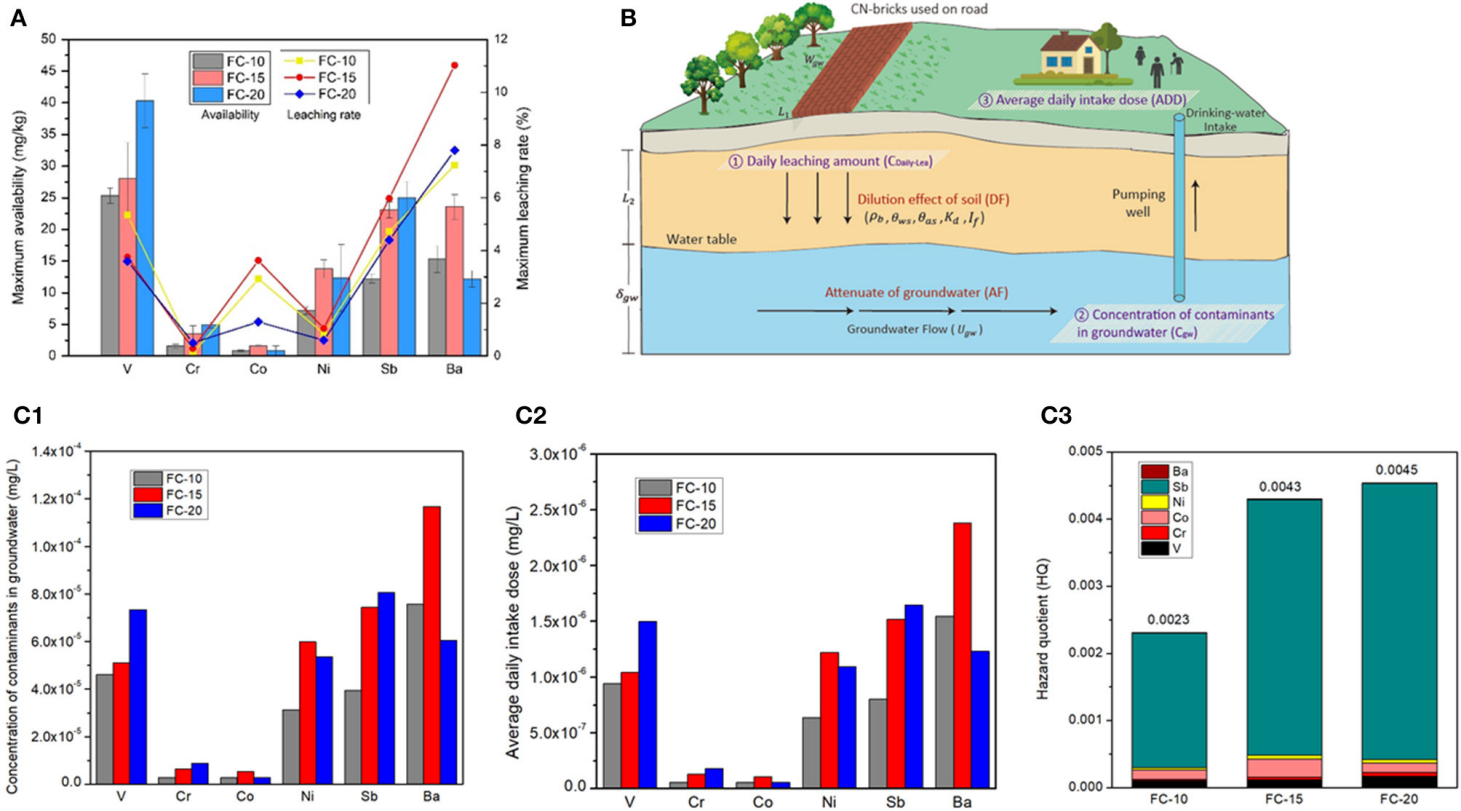
Maximum availability and maximum leaching rate **(A)**, exposure scenario analysis of CN-brick's utilization **(B)**, Concentration of contaminants in groundwater **(C1)**, Average daily intake dose **(C2)** and Hazard quotient **(C3)** of CN-bricks (FC-10, FC-15 and FC-20).

An exposure scenario analysis of CN-bricks is depicted in Figure 7B, which generally includes: (1) determination of the concentration of pollution source, which refers to the daily leaching amount (C_Daily−Lea_) of a CN-brick; (2) calculation of the concentration of contaminants in groundwater (C_gw_); and (3) human health risk assessment considering an average daily intake dose (ADD) of nearby residents and hazard quotient (HQ) of CN-bricks.

The maximum availability of a CN-brick was tested under EA NEN 7371. Figure 7A shows that the leaching level tested under NEN 7371 is significantly higher than that tested under TCLP, by almost three orders of magnitude. This is because the pH level maintained in the NEN 7371 test is more stable by continuously adding HNO_3_ in two leaching stages, which make it more ideal to represent the long-term leaching behavior of materials (Youcai and Sheng, 2017). Moreover, the solidification effect of different metals is different, for example, the total content of Ni is higher than that of Sb (1,311 and 386 mg/kg), but the maximum availability of Ni is lower than Sb (12.35 and 24.99 mg/kg). But in general, a higher FCC content indicates a higher metal leaching risk of a CN-brick.

Afterward, the daily leaching amount (C_Daily−Lea_) is calculated from maximum availability in Equation (12):

(12) $$\begin{array}{r} C_{Daily-Lea}=\frac{C_{Max-Lea}\times p}{a\times d} \end{array}$$

where *p* is the density of a CN-brick, which is equal to 2,117 kg/m^3^; *a* is the service life of a CN-brick, which is equal to 30 years; *d* is the days of use in a year, which is set as 365 days. The concentration of contaminants in groundwater (*C*_gw_) was calculated from *C*_Daily−Lea_ in Equations (13)–(15):

(13) $$\begin{array}{r} C_{gw}=C_{Daily-Lea}/DAF \end{array}$$

(14) $$\begin{array}{r} DAF=DF\times AF \end{array}$$

(15) $$\begin{array}{r} DF=\frac{\left(H^{{}^{\prime }}\times \theta_{as}+\theta_{ws}+K_{d}\times \rho_{b}\right)\times \left(1+\frac{U_{gw}\times \delta_{gw}}{I_{f}\times W_{gw}}\right)}{\rho_{\text{b}}}\times \frac{L_{2}}{L_{1}} \end{array}$$

where DAF is the dilution attenuation factor. AF is selected as 1.0 since the maximum risk is considered. DF is calculated according to Equation (15) which is quoted from the Chinese standard HJ 25.3-2014 (HJ 25.3, 2014) and Wyoming Voluntary Remediation Program report (VRP, 2000). Equation (15) is widely utilized to represent the dilution effect of soil pollutants mitigating into groundwater. The reference values in the DF equation are listed in Supplementary Table 1, which are all quoted from the Chinese standard of risk assessment of contaminated sites (HJ 25.3, 2014), and the estimated DAFs are listed in Table 4. The values of *C*_gw_ are then determined in Equation (13) and compared with the Chinese groundwater quality standard (GB/T 14848, 2017) and surface water quality standard (GB 3838, 2002; Table 4). The groundwater concentrations of six heavy metals range from 2.74E-06 to 1.17E-04 mg/L, which are all way below the regulatory limit of quality standards, indicating that CN-bricks will not pollute the groundwater to an unacceptable level.

**Table 4:** Maximum availability and daily leaching amount of CN-bricks and concentration of contaminants in groundwater.

**Samples**	**V**	**Cr**	**Co**	**Ni**	**Sb**	**Ba**
**Maximum availability of leaching (C**_**Max-Lea**_**), mg/kg**						
FC-10	25.34 ± 1.23	1.59 ± 0.28	0.84 ± 0.11	7.21 ± 0.61	12.21 ± 0.69	15.3 ± 2.13
FC-15	28.04 ± 5.67	3.57 ± 1.23	1.62 ± 0.19	13.79 ± 1.43	23.09 ± 1.28	23.58 ± 1.96
FC-20	40.31 ± 4.24	4.97 ± 0.53	0.85 ± 0.78	12.35 ± 5.28	24.99 ± 2.62	12.20 ± 1.30
**Daily leaching amount (C**_**Daily-Lea**_**), mg/L**						
FC-10	4.90E-03	3.07E-04	1.62E-04	1.39E-03	2.36E-03	2.96E-03
FC-15	5.42E-03	6.90E-04	3.13E-04	2.67E-03	4.46E-03	4.56E-03
FC-20	7.79E-03	9.61E-04	1.64E-04	2.39E-03	4.83E-03	2.36E-03
**Concentration of contaminants in groundwater (C**_**gw**_**), mg/L**						
FC-10	4.61E-05	2.82E-06	2.71E-06	3.13E-05	3.94E-05	7.57E-05
FC-15	5.10E-05	6.34E-06	5.22E-06	5.99E-05	7.45E-05	1.17E-04
FC-20	7.34E-05	8.82E-06	2.74E-06	5.36E-05	8.06E-05	6.04E-05
DAF	106.20	108.94	59.95	44.54	59.95	39.06
**Standard limits of groundwater and surface water's quality, mg/L**						
GB/T14848-2017 limits III	–	≤ 0.05	≤ 0.05	≤ 0.02	≤ 0.005	≤ 0.70
GB3838-2002	≤ 0.05	≤ 0.05	≤ 0.05	≤ 0.02	≤ 0.005	≤ 0.70

#### Human Health Risk Assessment

The final step of risk assessment is the human health risk assessment, which considers the hazards faced by nearby residents when they ingest pollutants through groundwater intake. The main exposure pathway discussed in this study is through drinking water, since other pathways (inhalation, skin, or food) can be far less harmful than a drinking water route. The daily intake of pollutants by an adult is calculated according to Equation (16), which is quoted from Risk Assessment Guidance for Superfund RAGS (1989) of USEPA:

(16) $$\begin{array}{r} ADD=\frac{C_{i}\times IngR\times EF\times ED}{BW\times AT} \end{array}$$

where ADD is the average daily intake dose of an element *i* (mg/kg · day), and *C*_*i*_ is the concentration of a metal *i* in drinking water, which equals to *C*_gw_. IngR is the ingestion rate; EF is exposure frequency; ED is the exposure duration; BW is the body weight; and AT is the average exposure time. The units and values of the above-mentioned parameters are summarized in Supplementary Table 2, and the results of ADD results are shown in Figure 7C. Then, the HQ is authenticated based on the ADD level. According to US Environmental Protection Agency (USEPA), chemicals are classified as carcinogens and non-carcinogens, the risk of which are, respectively, represented as cancer risk (CR) and HQ. According to USEPA, integrated risk information system (IRIS, 1989), and Chinese Standard HJ 25.3-2014 (HJ 25.3, 2014), only arsenic (As) in heavy metals is considered as carcinogen, all the six metals discussed earlier are not included. Thus, only non-carcinogenic risk is considered in this research, and the HQ is calculated as follows (RAGS, 1989):

(17) $$\begin{array}{r} HQ=\frac{ADD}{RfD_{i}} \end{array}$$

where RfD_i_ is the referenced dose that is interpreted as “an exposure level that is likely to be without an appreciable risk of deleterious effects during a lifetime,” and the RfD_*i*_ value of six elements is listed in Supplementary Table 3 according to IRIS (1989). The RAGS guidance indicates that a HQ index lower than 1.0 shows an acceptable risk, whereas the potential of non-carcinogenic risk for human may occur when HQ > 1.0 (RAGS, 1989). To assess the overall non-carcinogenic risk of a CN-brick, the HQs of each contaminant should be summed up and presented as hazard index (HI).

(18) $$\begin{array}{r} HI=\sum_{1}^{\text{i}}HQ \end{array}$$

The non-carcinogenic risk assessment results of three different CN-bricks are summarized in Figure 7C. CN-bricks with different proportions of a spent FCC catalyst have different HI, the more the spent FCC catalyst were added, the higher the HI will be, which are 0.0023, 0.0043, and 0.0045, respectively. All the HIs of three CN-bricks are all significantly lower than 1.0, proving that when a person is exposed to this dose level over a lifetime, no non-carcinogenic effects is expected to be detected in his/her body over a lifetime. In sum, CN-brick will not pose a threat to environment and human bodies, which can be utilized on road pavement for 30 years without environmental hazard. The environmental risk assessment result in this study can provide a technical basic for the assessment of the utilization of a CN-brick on roads and embankments in the future. For future application of CN-bricks on road, the groundwater detection well should be set nearby for regular detection of groundwater quality. In the process of using pavement materials, operators should take necessary protection to avoid working in windy weather.

It deserves attention from Figure 7C that the HQ of Sb accounts for 86.9, 88.4, and 90.6% of whole HI of FC-10, FC-15, and FC-20, respectively, which constitute a main environmental risk of CN-bricks. The reference dose of Sb, 0.0004 mg/kg · d, is much lower than other metals (Supplementary Table 3). Sb belongs to the same group as As and have a potential carcinogenic risk, so it has been listed as priority pollutant by US EPA and hazardous wastes by European Union. Sb is the third most abundant element in CN-bricks (Figure 6) due to the addition of Sb-type Ni passivator in the sampling unit, which is used to suppress the negative effects of metal Ni. Element Co also has a strict reference dose, 0.0004 mg/kg · d, but the daily intake dose of Co is way lower than Sb (Figure 7C), making it accounting for only a small part of the HI. For future production and utilization of a CN-brick, a raw material of spent FCC catalyst should be cautiously selected. The catalyst coming from units without a Sb-type Ni passivator should be given priority. For catalytic cracking unit in refinery plants, innoxious metal passivators without Sb should be preferred according to the result of environmental risk assessment in this study.

The environmental risk assessment of a CN-brick entails certain uncertainty, which is inevitable in quantitative risk assessment. In the calculation of contaminants in groundwater and ADD, the actual measured data should be given priority, so the reference date may induce a certain deviation. However, there is no absolute accurate assessment, the exposure analysis in this study is based on a well-defined calculation and could represent the actual environmental risk of CN-bricks.

## Conclusion

This paper focuses on utilizing a spent FCC catalyst as a partial replacement of a fine aggregate in the production of a NS-brick. 20% cement +Na_2_SO_4_ is proven to be an optimal binder system, which could improve the compressive strength up to 42.3 MPa. Best grain size distribution occurs at 20% spent FCC catalyst + 45% gravel, which could achieve the lowest porosity and water absorption rate, yet a higher spent FCC catalyst proportion would deteriorate the property of a CN-brick. The main hydration products of a CN-brick are C-S-H and AFt. Alkali activators could create an alkaline environment that dissolve more [SiO_4_]^4−^ and [AlO_4_]^5−^ from the raw materials, and Na_2_SO_4_ can particularly promote the formation of a needle-like AFt to a large extent. The TCLP test shows that V, Cr, Co, Ni, Sb, and Ba are the characteristic pollutants of CN-bricks. An exposure scenario analysis based on the NEN 7371 leaching test works out a final HI from 0.0023 to 0.0045 for a CN-brick, indicating its acceptable risk for environment and humans. Thus, it can be said that producing NS-bricks from a spent FCC catalyst is technically feasible and environment friendly.

## Data Availability Statement

The original contributions presented in the study are included in the article/Supplementary Material, further inquiries can be directed to the corresponding author/s.

## Author Contributions

SZ, SF, and HZ came up with the conception and directed the study. DZ, ZL, and ZZ carried out the experiments. DZ and ZL performed the statistical analysis. DZ finished the manuscript. All authors have revised the final version of the manuscript and approve of submission.

## Conflict of Interest

The authors declare that the research was conducted in the absence of any commercial or financial relationships that could be construed as a potential conflict of interest.
