# Supplementary material for: Utilization of Spent FCC Catalyst as Fine Aggregate in Non-sintered Brick: Alkali Activation and Environmental Risk Assessment
Source: Front Chem. 2021 Apr 26;9:674271. doi: 10.3389/fchem.2021.674271 (PMC8108481; doi:10.3389/fchem.2021.674271)
Supplement: Supplementary file 1 [file Data_Sheet_1.docx]

# Data recovered from BindingDB

Table S1: Compounds deposited in BindingDB tested as treatment for Leishmaniasis

| Drug ID | Paper | ChEMBL ID for Assay to Leishmania species | Target Described |
| --- | --- | --- | --- |
| 912 | 10.1021/jm0493717 | ChEMBL_430130 |  |
| 6644* | 10.1021/jm901059x | ChEMBL_596055 | LmjF.23.0270 |
| 12576* | 10.1021/jm030084x 10.1021/jm0582625 10.1021/jm040209d | ChEMBL_69823 ChEMBL_330072 ChEMBL_302625 | LmjF.22.1360 |
| 12578* | 10.1021/jm030084x 10.1021/jm040209d | ChEMBL_69823 ChEMBL_302625 | LmjF.22.1360 |
| 12581* | 10.1021/jm040209d | ChEMBL_302625 | LmjF.22.1360 |
| 14487* | 10.1021/jm00019a014 10.1021/jm00047a017 10.1021/jm9802620 10.1016/s0960-894x(98)00635-0 | ChEMBL_72881 ChEMBL_72880 ChEMBL_72770 ChEBML_72905 | LmxM.34.4750(GAPC) |
| 18050* | 10.1021/jm300563f 10.1021/jm901059x 10.1021/jm00390a017 | ChEMBL_876348 ChEMBL_596055 ChEMBL_55096 | LmjF.23.0270 |
| 18069* | 10.1021/jm981130+ | ChEMBL_55095 | LmjF.06.0860 |
| 18512* | 10.1021/jm981130+ | ChEMBL_55095 | LmjF.06.0860 |
| 25313* | 10.1021/jm040209d | ChEMBL_302625 | LmjF.22.1360 |
| 25400* | 10.1021/jm9802620 | ChEMBL_72770 | LmxM.34.4750(GAPC) |
| 27507* | 10.1016/j.ejmech.2012.11.014 | ChEMBL_936768 | LinJ.26.0200 |
| 28422* | 10.1021/jm00079a007 | ChEMBL_161418 |  |
| 30705* | 10.1021/jm1010572 | ChEMBL_702837 | LmjF.23.0270 |
| 81747* | 10.1111/j.1747-0285.2008.00729.x |  | LtaP25.1210 (Tubulin alpha chain) |
| 84608 | 10.1016/j.ejmech.2014.12.051 | ChEMBL_1470027 ChEMBL_1470026 | LmjF.06.0860 |
| 50028122* | 10.1021/jm901059x | ChEMBL_596055 | LmjF.23.0270 |
| 50035218* | 10.1111/j.1747-0285.2008.00729.x |  | LtaP25.1210 (Tubulin alpha chain) |
| 50080390* | 10.1021/jm9802620 | ChEMBL_72770 | LmxM.34.4750(GAPC) |
| 50081908* | 10.1021/jm981130+ | ChEMBL_55095 | LmjF.06.0860 |
| 50081921* | 10.1021/jm981130+ | ChEMBL_55095 | LmjF.06.0860 |
| 50085667* | 10.1021/jm9802620 | ChEMBL_72770 | LmxM.34.4750(GAPC) |
| 50090054* | 10.1021/jm00390a017 | ChEMBL_55096 | LmjF.06.0860 |
| 50090067* | 10.1021/jm00390a017 | ChEMBL_55096 | LmjF.06.0860 |
| 50097886* | 10.1021/jm030084x | ChEMBL_69823 | LmjF.22.1360 |
| 50135822 | 10.1021/jm030084x | ChEMBL_69823 | LmjF.22.1360 |
| 50241166* | 10.1021/jm9802620 10.1016/s0960-894x(98)00635-0 | ChEMBL_72770 ChEBML_72905 | LmxM.34.4750(GAPC) |
| 50286441 | 10.1021/jm0505765 | ChEMBL_336666 |  |
| 50291777* | 10.1021/jm00390a017 | ChEMBL_55096 | LmjF.06.0860 |
| 50291796* | 10.1021/jm00390a017 | ChEMBL_55096 | LmjF.06.0860 |
| 50291799* | 10.1021/jm00390a017 | ChEMBL_55096 | LmjF.06.0860 |
| 50303504 | 10.1021/jm901059x | ChEMBL_596055 | LmjF.23.0270 |
| 50335098 | 10.1021/jm1010572 | ChEMBL_702837 ChEMBL_702836 | LmjF.23.0270 |
| 50378739 | 10.1021/jm00079a007 | ChEMBL_161421 |  |
| 50408666* | 10.1021/jm9802620 | ChEMBL_72770 | LmxM.34.4750(GAPC) |
| 50465949 | 10.1021/acs.jmedchem.8b01671 | CHEMBL4276822 | LinJ.11.1100 |
| 50465948 | 10.1021/acs.jmedchem.8b01671 | CHEMBL3629567 | LinJ.11.1100 |
| 213825 | 10.1021/acs.jmedchem.8b01671 |  | LinJ.11.1100 |
| 50276214 | 10.1021/acs.jmedchem.8b01671 | CHEMBL4129161 | LinJ.11.1100 |
| 50363766 | 10.1016/j.bmc.2012.01.009 | CHEMBL242165 | LinJ.05.0350 |

* compounds were recovered for our analysis due to high affinity with targets in other species

# Differentially expressed genes


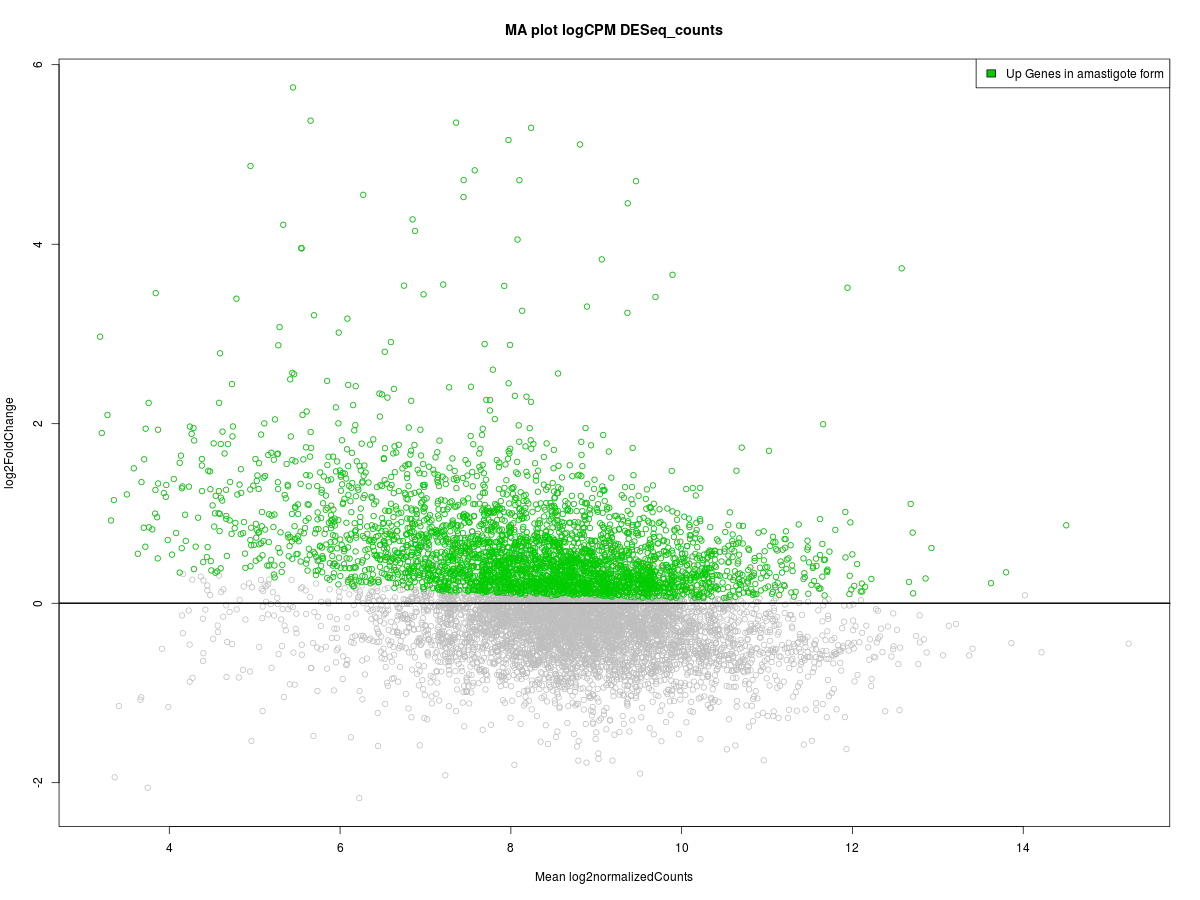


MAplot of differentially expressed genes. The up regulate genes in the amastigote form are marked in green.

3 Categorization of GO terms predicted for *Leishmania* *sp.*


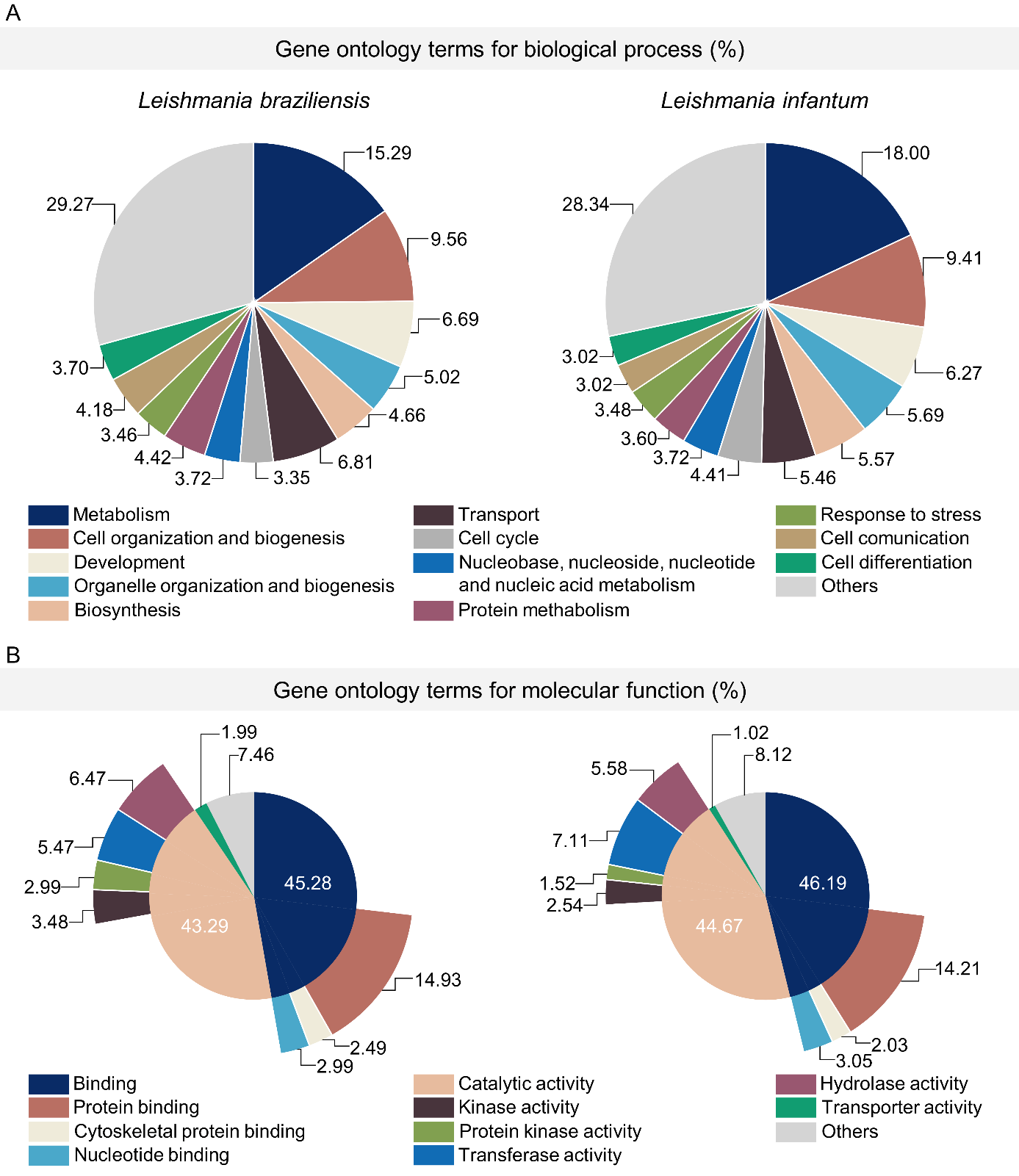


The GO terms predicted using the blast2Go tool for *L. braziliensis* (graphics on the left) and *L. infantum* (graphics on the right) categorized according to the most present terms. (A) categories associated with biological processes; (B) categories associated with molecular functions*.*
